# Cost saving in implementing ERAS protocol in emergency abdominal surgery

**DOI:** 10.1186/s12893-024-02345-y

**Published:** 2024-02-22

**Authors:** Pietro Bisagni, Vera D’Abrosca, Vincenzo Tripodi, Francesca Teodora Armao, Marco Longhi, Gianluca Russo, Michele Ballabio

**Affiliations:** 1 Department of Surgery, Ospedale Maggiore di Lodi, Viale Savoia 1, 26900 Lodi, Italia; 2 Department of Emergency, Ospedale Maggiore di Lodi, Lodi, Italy; 3 Università degli Studi Statale di Milano, Milano, Italy

**Keywords:** General surgery, Emergency surgery, Visceral surgery, ERAS, Acute care surgery

## Abstract

**Introduction:**

ERAS (Enhanced Recovery After Surgery) protocol is now proposed as the standard of care in elective major abdominal surgery. Implementation of the ERAS protocol in emergency setting has been proposed but his economic impact has not been investigated. Aim of this study was to evaluate the cost saving of implementing ERAS in abdominal emergency surgery in a single institution.

**Methods:**

A group of 80 consecutive patients treated by ERAS protocol for gastrointestinal emergency surgery in 2021 was compared with an analogue group of 75 consecutive patients treated by the same surgery the year before implementation of ERAS protocol. Adhesion to postoperative items, length of stay, morbidity and mortality were recorded. Cost saving analysis was performed.

**Results:**

50% Adhesion to postoperative items was reached on day 2 in the ERAS group in mean. Laparoscopic approach was 40 vs 12% in ERAS and control group respectively (p ,002). Length of stay was shorter in ERAS group by 3 days (9 vs 12 days p ,002). Morbidity and mortality rate were similar in both groups. The ERAS group had a mean cost saving of 1022,78 € per patient.

**Conclusions:**

ERAS protocol implementation in the abdominal emergency setting is cost effective resulting in a significant shorter length of stay and cost saving per patient.

## Introduction

ERAS (Enhanced Recovery After Surgery) protocol has been introduced as standard of care in elective major abdominal surgery [1]. The economic effect of this protocol has been deeply investigated particularly in the field of elective colorectal surgery in which ERAS implementation has been associated with shorter length of stay (LOS) and reduction of postoperative complications rate [2–4].

Guidelines for the use of ERAS protocol in the abdominal emergency setting have been recently proposed [5]. Preliminary results in performing ERAS protocol in the field of intestinal obstruction and perforated peptic ulcers have been published [6, 7].

Safety and feasibility of the ERAS protocol in major gastrointestinal emergency surgery has been demonstrated in a recent paper. Laparoscopy, avoiding abdominal drainage and intraoperative fluid overload have been associated with faster recovery [8].

The economic impact of ERAS in the emergency setting has not been estimated.

Aim of this study was to analyze the cost effectiveness in implementing ERAS protocol in emergency abdominal gastrointestinal surgery in a single institution.

## Methods

In 2021 in Lodi Maggiore Hospital ERAS protocol has been implemented in gastrointestinal emergency surgery as one of the centers participating to a multicenter study.

Inclusion criteria were:

- Unscheduled abdominal surgery (bowel resection with or without anastomosis, hollow viscus repair, enteric bypass or adhesiolysis, in the presence of peritoneal contamination or bowel obstruction)

Exclusion criteria:

- Age < 18
- Minor abdominal surgery (appendectomy, cholecystectomy, simple abdominal wall or hernia repair)
- Emergency abdominal surgery due to complications of elective surgery or endoscopic/ radiologic procedures
- Pregnancy
- Refuse to participate or refuse data collection.
- Patients treated by damage control strategy with open abdomen with ICU stay more then 72 hrs

This study protocol was approved by the Ethical Committee of the promoting center (n. 0,012,747 08/10/2020) and was registered on clinical trial.gov (identifier NCT04648644) [8].

Control group was constituted by an analogue cohort of consecutive patients treated in the same hospital selected by the same criteria the year before implementation of ERAS protocol in elective surgery.

Informed consent was obtained from all subjects or their legal guardians.

Both groups were compared for age, sex, BMI, Charlson Comorbidity Index, time to surgery, Lactate and Hemoglobin value.

Intraoperative data about type and duration of surgery, surgical technique and anesthesiologic procedures were recorded as well.

Data about nasogastric tube and urinary catheter removal, oral and food intake, mobilization > 4 hrs and i.v. fluid stop postoperative day were collected based on clinical record.

Postoperative complications were graded according to Clavien Dindo scale [9] in both groups. Length of stay, 30-day mortality and readmission rate were also measured.

Primary end point of this study was assessment of cost saving differences between the two groups.

### Confront statistics

Data from both groups were analyzed using IBM SPSS Statistics software (2020. IBM SPSS Statistics for Windows, Version 27.0. Armonk, NY: IBM Corp.). In confronting the 2 groups two-tailed Mann – Whitney or Student’s t test whereas Chi square and Fisher exact test were used confronting categorical variables. Nonparametric Bootstrap t test was used for cost analysis [10]. P value <0.05 was considered statistically significant.

### Cost analysis

All costs have been calculated in Euros. Arithmetical mean cost per patient has been considered effective.

Single Day of hospitalization cost was obtained considering all fixed costs and all the ordered medicaments and devices and dividing it for the total numbers of days of hospitalization.

Costs of surgical procedures have been estimated in terms of duration of surgery and use of laparoscopy. Costs of complications have been calculated in terms of days of ICU, cost of radiological or surgical procedures when performed, cost of medication and unplanned clinic or ER admission.

The cost of ERAS protocol implementation has been estimated based on preoperative and intraoperative anesthesia.

All costs were normalized at the 2022 costs analysis.

## Results

Eighty consecutive patients treated by ERAS in emergency setting were compared to a similar group of 75 consecutive patients treated in 2017 (year before introduction of ERAS protocol in elective surgery in our institution). The groups were similar in age, sex, BMI, Charlson comorbidity index, preoperative lactate and Hb. The characteristics of both groups are reported in Table 1.

**Table 1:** Preoperative data

		***ERAS group (n: 80)***	***Control group (n:75)***	***p-value***
Age (yr)		69 (st dv 16)	72 (st dv 16)	,281*
Male number		33 (41%)	33 (44%)	,748°
BMI (Kg/m^2^)		25 (st dv 5)	24 (st dv 5)	,281*
Time to surgery (hr)		17 (st dv 2)	14 (st dv 3)	,586*
Charlson Com Index				,795^
	0-2	19 (23,7%)	23 (30,7%)	
	3-4	23 (28,7%)	17 (22,7%)	
	5-10	38 (47,6%)	35 (46,6%)	
Lactate (mmol/L)		2.08 (st dv 2)	2,75 (st dv 2)	,441*
Hb (mg/dL)		12.8 (st dv 3)	12,7 (st dv 2)	,427*

^*^ Student’s T test, ° Fisher Exact test, ^ Chi Square test

### Intra and postoperative results

ERAS group had significant compliance to intra and postoperative items comparing to control group as reported in Table 2. The highest adherence was obtained on depth of anesthesia monitoring, neuromuscolar blockade monitoring and prevention on nausea and vomiting in intraoperative items, whereas in postoperative items the most significant differences with the control group were on nasogastric tube and urinary catheter removal, oral fluid and solid intake.

**Table 2:** Intraoperative and Postoperative adherence ERAS item

	***ERAS group (n 80)***	***Control Group (n 75)***	***p-value***
***Intraoperative items***			
Depth of anesthesia monitoring (entropy)	80 (100%)	74 (98,7%)	,484*
Neuromuscolar blockade monitoring	80 (100%)	71 (94,7%)	,053*
**PONV (prevention on nausea and vomiting)**	**63 (78,7%)**	**19 (25,3%)**	,000*
**Invasive arterial pressure monitoring**	**9 (11,2%)**	**1 (1,3%)**	,018*
**Inotropes/vasopressor**	**22 (27,5%)**	**8 (10,75)**	,004*
Fluid management	+ 1484 ml (st dv 879)	+ 744 ml (st dv 1246)	,095^
***Postoperative items - 50% adherence- day***			
**Nasogastric tube removal**	**2**	**6**	**,001°**
**Oral fluid intake**	**2**	**4**	**,000°**
Mobilization > 4 hrs	2	3	,579°
**Urinary catheter removal**	**2**	**5**	**,001°**
**Solid food intake**	**3**	**5**	**,000°**
**I.v. fluid stop**	**5**	**7**	**,004°**

^*^Fisher exact test, ° Chi square test, ^ U Mann-Whitney test

Data about intraoperative management are reported in Table 3. Laparoscopy number was significantly higher in ERAS group with a conversion rate of 50%. Both laparoscopic and open surgery were performed by the same surgical team; in the 4 years observed, surgical team turnover was 25% (retirement and new hire).

**Table 3:** Data about surgery

		***ERAS group (n 80)***	***Control group (n 75)***	***p-value***
Duration of Surgery (min)		124 (st dv 59)	128 (st dv 60)	,683*
Surgery				,099^
	Hollow viscus repair	8 (10%)	3 (4%)	
	Adhesiolysis	26 (32,5%)	20 (26,7%)	
	Resection with anastomosis	30 (37,5%)	31 (41,3%)	
	Resection without anastomosis	13 (16,3%)	21 (28%)	
	Bypass	3 (3,7%)	0	
**Surgical Technique**				**,002^**
	**Open**	**48 (60%)**	**66 (88%)**	
	**VLS**	**16 (20%)**	**1 (1,3%)**	
	**VLS conv to open**	**16 (20%)**	**8 (10,7%)**	
Lactate end of surg (mmol/L)		1,69 (st dv 1)	2,25 (st dv 1)	,989’
**Drainage**		**65 (81,2%)**	**71 (94,7%)**	**,014°**

^*^U Mann-Whitney test, ^ Chi square test, ° Fisher exact test, ‘ Student’s T test

Surgical procedures were 8 vs 3 hollow viscus perforations repair and 26 vs 20 adhesiolysis in the ERAS and control group respectively: whereas resections were 30 vs 31 with anastomosis and 13 vs 21 without anastomosis in the ERAS and Control group respectively. 3 bowel bypasses were performed in the ERAS group.

**Fig. 1:**
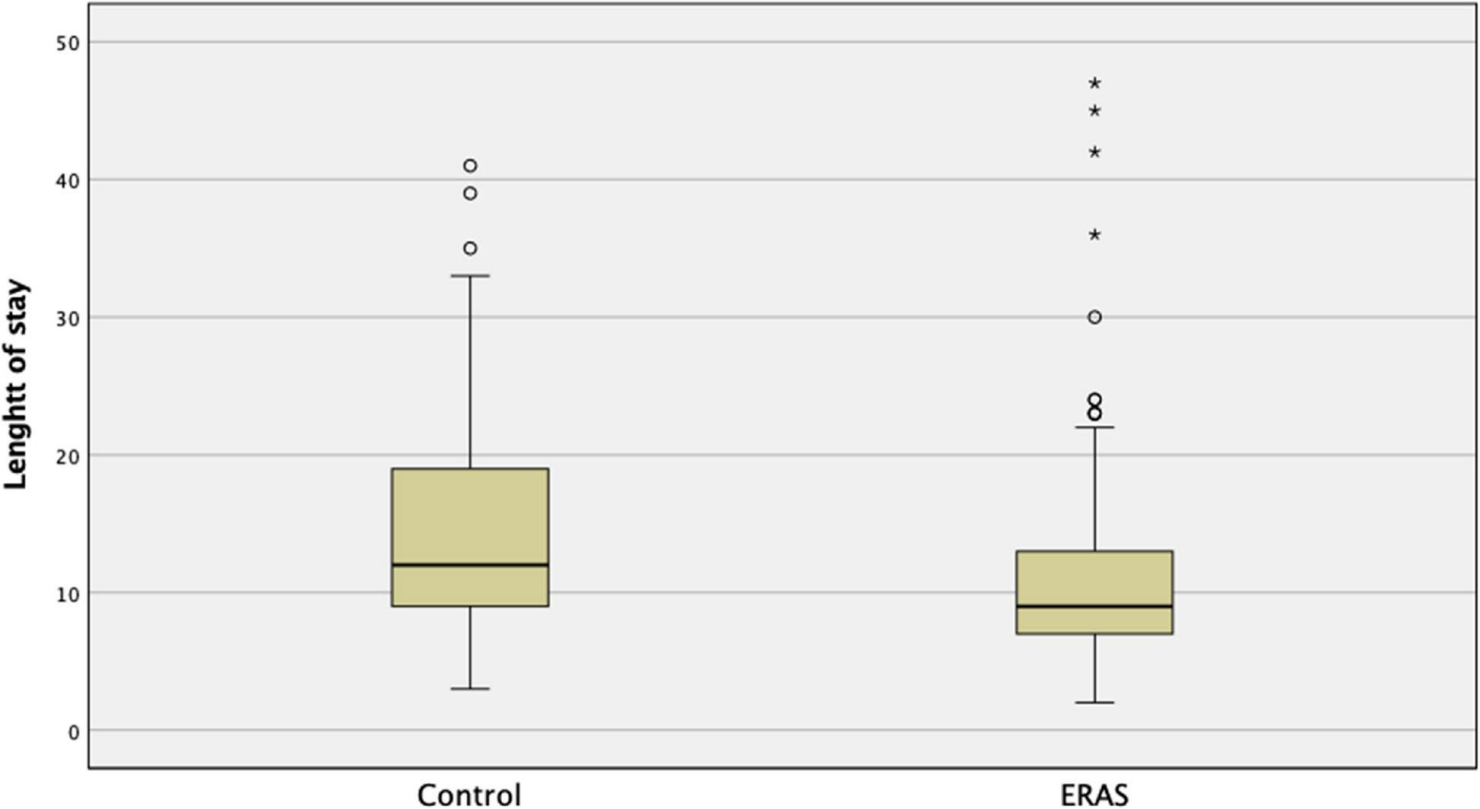
Length of Stay

**Fig. 2:**
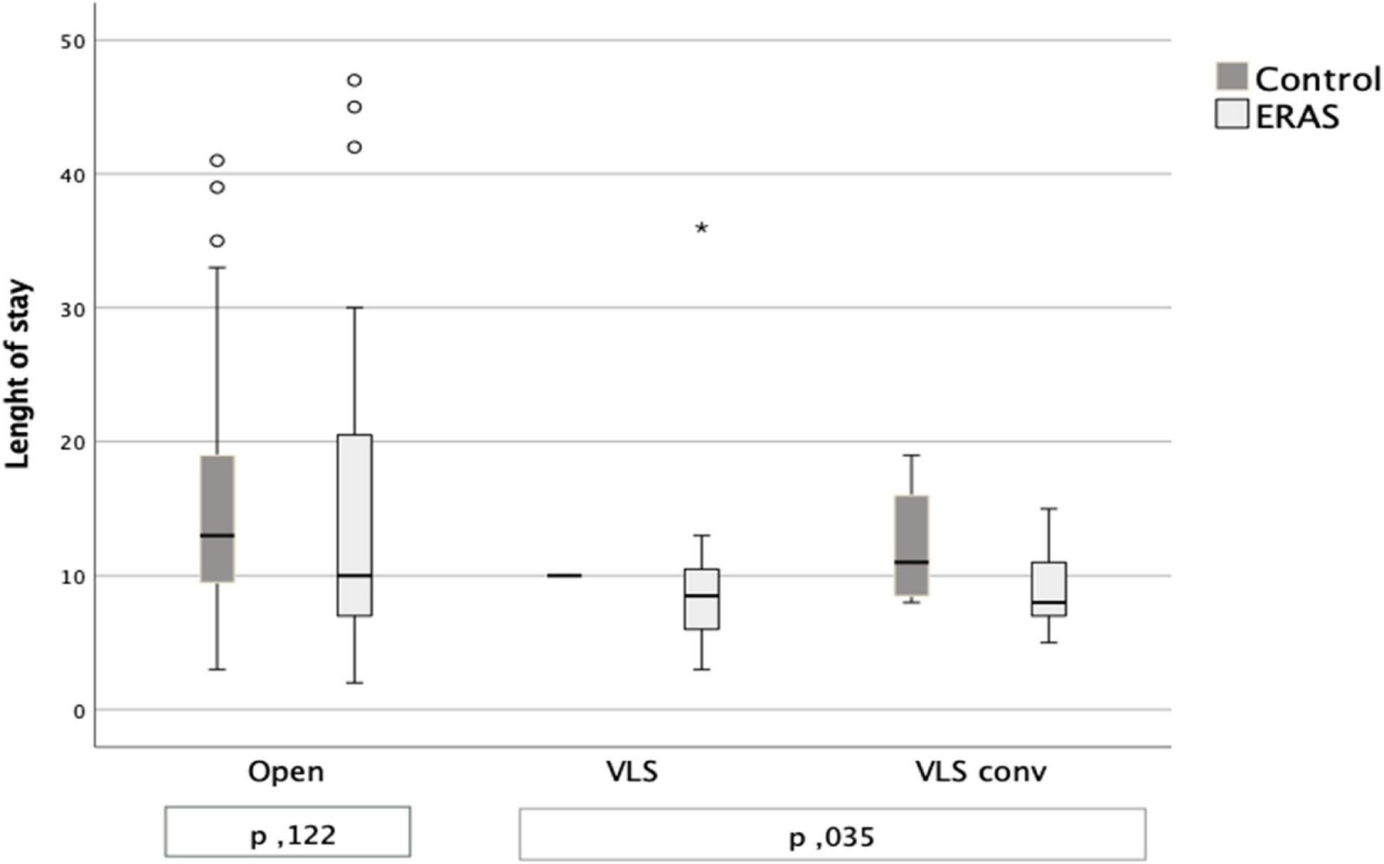
Length of stay per surgical technique. U Mann Whitney test

Length of stay was significantly faster in the ERAS group (Fig. 1) and seemed to be different regardless the surgical technique (Fig. 2).

Complications, mortality and readmission rate were similar in both groups and are reported in Table 4.

**Table 4:** Postoperative results

		***ERAS group (80)***	***Control Group (75)***	***p-value***
**Lenght of Stay (median)**		**9 (2 - 47)**	**12 (3 – 41)**	,002*
	Open surg subgroup (48 vs 66)	10 (2 – 47)	13 (3 – 41)	,122*
	VLS surg subgroup (32 vs 9)	8 (3 – 36)	11 (8 – 19)	,035*
Complication CD class* [9]				,981°
	I	10 (12,5%)	11 (14,7%)	
	II	19 (23,8%)	17 (22,7%)	
	IIIa	2 (2,5%)	1 (1,3%)	
	IIIb	5 (6,3%)	4 (5,3%)	
	IV-V	8 (10%)	6 (8%)	
Surgical Site Infection		19 (23,7%)	9 (12%)	,068°
Anastomotic Leakage		3 (3,7%)	2 (2,7%)	,729°
Respiratory infection		7 (8,7%)	6 (8%)	,912°
Urinary tract infection		4 (5%)	3 (4%)	,796°
Cardiovascular complication		8 (10%)	8 (10,7%)	,864°
Rehospitalizations		1 (1,2%)	1 (1,3%)	,923°
30 days Mortality rate		8 (10%)	6 (8%)	,664°

^*^U Mann Whitney test, ° Chi square test

### Cost analysis

Preoperative costs were similar in both groups except for the cost of implementing ERAS protocol in emergency surgery in terms of training and auditing for surgeons, anesthesiologist and nurses.

One day of staying in the surgical unit had a cost of € 542,26 (Table 5).

**Table 5:** Calculation of single day cost in general surgery ward in Lodi Maggiore Hospital in 2022

**Hospital Unit**	**Year**	**Item of Costs**	**Cost in € per year**
General Surgery	2022	Medicaments	17098,63
General Surgery	2022	Staff	2575885,49
General Surgery	2022	Hospital Facilities	413453,71
General Surgery	2022	Equipment	73888.30
General Surgery	2022	Administrative	646065,20
		TOTAL	3726391,33
General Surgery	2022	Days of hospitalizations	6872
General Surgery	2022	Single Day Cost (except OR Costs)	542,26

OR costs have not been considered in this computation

Intraoperative costs were significantly higher in the ERAS group due to increased number of laparoscopies. Anesthesia costs were higher, although not statistically significant, in the ERAS group.

ERAS group had significant cost saving in the staying costs. Mean complications costs in term of redo surgery, ICU staying, readmission, ambulatory visits and medications have been calculated (Table 6).

**Table 6:** Costs per patients’ means (2022 Costs)

	***ERAS group (80)***	***Control group (75)***	p*
Anesthesia costs	1019,90 € (929,58-1127,04)	881,19 € (791,80-983,57)	,065
Intraoperative costs and laparoscopy	2903,11 € (2648,64-3168,47)	2481,18 € (2196,31-2789,90)	,048
ICU and Complication costs	5601,72 € (3551,74-7779,01)	5244,94 € (3265,58 -7410,86)	,821
Length of stay and others postop costs	6523,87 € (5511,33-7673,30)	8644,41 € (7182,91-10354,58)	,048

Values are means derived from 2000 non-parametric bootstrap replicate, with 95% confidence intervals

^*^p value calculated by nonparametric Bootstrap t test

Costs item’s mean differences per patient such as preoperative, anesthesia, intraoperative and postoperative have been reported. The total cost saving in this study was € 1022,78 per patient (Table 7).

**Table 7:** Summary of mean cost saving per patient

	***ERAS***	***Control***	***Differences ERAS - Control***
**Protocol implementation costs**	**180,34 €**	**-**	**+180,34 €**
**Anesthesia costs**	**1019,90 €**	**881,19 €**	**+138,71 €**
**Intraoperative costs and laparoscopy**	**2903,11 €**	**2481,18 €**	**+421,93 €**
**ICU and Complication costs**	**5601,72 €**	**5244,94 €**	**+356,78 €**
**Length of stay and others postop costs**	**6523,87 €**	**8644,41 €**	**-2120,54 €**
**Total cost minimization**			**1022,78 €**

## Discussion

This is a retrospective cohort analysis of cost saving in implementing ERAS protocol in abdominal emergency surgery in a single institution.

Safety and feasibility of ERAS protocol in emergency major abdominal surgery have been recently described [8, 11, 12].

In this study ERAS group had significant compliance to postoperative items as reported in Table 2. There is still space for improvement about some of the intraoperative items (fluid management, drainage and laparoscopy) when comparing to colorectal ERAS protocol [1]. Thus, leading to possible better cost saving results.

An estimation of the economic impact in treating patients by ERAS in the emergency setting has not been published. The present study shows a cost saving of € 1022,78 per patient in a single center experience.

Generally, cost saving analysis lack of considerations of all costs for patient [13]. In the present study the cost of implementation as well as the cost saving on length of stay and complications were considered.

A shorter length of stay might in part explain the cost saving after the introduction of a specific clinical pathways as previously described [14]. In our experience 3 days was the median of shortening the hospital stay in the ERAS group (Fig. 1). This result, which counts for about 25% of the median length of stay, is not explained just with a difference in the discharge management over time between the 2 period of observation.

The difference in length of stay in the ERAS vs control group was observed regardless the surgical technique gaining a statistical significance in the laparoscopic intention to treat subgroup (Fig. 2).

In fact, the impact of length of stay in cost saving has been broadly discussed because the cost of a single day of hospitalization is different in the first postoperative course respect the end of the hospital stay [15].

The way we used to obtain the medium cost of a single postop day considering all the fixed costs and all the costs of ordered medicaments and devices in a single ward and dividing it for the numbers of days of hospitalization should be an adequate approximation of a real median cost per day per patient (Table 5).

The cost of operatory room is practically not affected by the implementation of ERAS the only differences we found were about numbers of laparoscopy and costs of anesthesia but the mean OR time per patient was similar in both groups.

Laparoscopy seems to have an important role in shortening hospital stay and is part of the ERAS pathway as described in previous paper [16–18]. Looking at the intention to treat, 40% of attempted laparoscopy in the ERAS group with a conversion rate of 50% versus 12% in the control group with 89% of conversions to open surgery were observed.

In the present experience the most important factor in cost saving is represented by the difference in length of stay but we certainly agree that complications have an important impact on costs [19].

The central point is whether a shorten length of stay in the ERAS group is due to ERAS protocol implementation or is a consequence of an increased rate of laparoscopic approach.

To answer this question, we should first consider that the difference in length of stay is present in the open approach group too even if not statistically significant (Fig. 2); therefore it seems to depend more on the ERAS protocol implementation.

On the other hand, laparoscopic approach is now considered a central point in intraoperative items of implementing ERAS protocol specifically in the emergency setting [6, 8, 11]. According with this we judge this length of stay difference as a results of ERAS protocol implementation.

In this series no significant differences in morbidity and readmissions rate between the 2 groups were observed. These findings are consistent with previous reports regarding surgical complications in implementing ERAS pathway [1–4]. On the other hand, the rate of complication might be explained in part considering baseline comorbidities of the patients treated, and in part it might be related to the partial number of items of the ERAS pathway achieved [1, 20].

Surgical site infections shows a trend in favor of the control group despite lower rate of laparoscopy. This trend, even if not statistically significant, might be in part explained by an increased number of hollow viscus sutures in the ERAS group.

This study presents several limitations, first is a retrospective cohort study and the 2 groups were recruited in a 4-year period, allowing some possible bias due to differences in treatments among the 2 periods and surgical teams turnover.

Second is a single center study which cares advantages in confronting the costs of complications and implementations but, of course, represents a limitation about the sample size.

Conversely this is the first analysis of costs saving about implementing ERAS in emergency surgery. Other larger and powerful studies will be mandatory to validate those preliminary results.

In conclusion this study supports implementation of ERAS protocol in emergency setting in a cost analysis point of view. In the present cohort of patients, implementing ERAS pathway in emergency abdominal surgery resulted in an average cost saving of € 1022,78 per patient.

## Data Availability

All data generated or analyzed during the current study are available from the corresponding author on reasonable request.
